# Definitive Radiotherapy versus Postoperative Radiotherapy of Patients with Oro- and Hypopharyngeal Cancer: Impact of Prognostic Factors

**DOI:** 10.1155/2012/391917

**Published:** 2012-01-18

**Authors:** Volker Rudat, Salia Ahmet-Osman, Oliver Schramm, Andreas Dietz

**Affiliations:** ^1^Department of Radiation Oncology, University of Heidelberg, Heidelberg 69120, Germany; ^2^Department of Radiation Oncology, Saad Specialist Hospital, Al-Khobar 31952, Saudi Arabia; ^3^Department of Head and Neck Surgery, University of Leipzig, Leipzig 04103, Germany

## Abstract

*Purpose*. To compare the impact of prognostic factors of patients treated with definitive radio(chemo)therapy versus patients treated with surgery and postoperative radiotherapy for squamous cell carcinoma of the oro- and hypopharynx. *Patients and Methods*. 162 patients treated with definitive radiotherapy and 126 patients treated with postoperative radiotherapy were retrospectively analysed. The impact of the prognostic factors gender, age, total tumor volume (TTV), pre-radiotherapy hemoglobin level (Hb-level), tumor site, T- and N-classification, radiotherapy interruptions >5 days, radiotherapy versus simultaneous radiochemotherapy, R-status and time interval between surgery and radiotherapy were investigated. *Results*. The median follow-up time for the censored patients treated with definitive radio(chemo)therapy was 28.5 months and for postoperative radiotherapy 36.5 months. On univariate analysis, the TTV, Hb-level, and simultaneous radiochemotherapy had a significant impact on the survival of patients treated with definitive radio(chemo)therapy. For patients treated with postoperative radiotherapy, only the TTV showed a statistical trend for the survival (*P* = 0.13). On multivariate analysis, the TTV and simultaneous radiochemotherapy maintained their statistical significance for patients treated with definitive raditherapy, and the TTV, the statistical trend for patients treated with postoperative radiotherapy (*P* = 0.19). *Conclusions*. The TTV was the predominant prognostic factor for both, patients treated with definitive or postoperative radiotherapy.

## 1. Introduction

Squamous cell carcinoma of the head and neck (SCCHN) is the fifth most common neoplasm with an estimated annual global incidence of more than 500,000 cases diagnosed worldwide [1]. The treatment is usually interdisciplinary and mainly involves surgeons, radiation oncologists, medical oncologists, clinical nurse specialists, speech and language specialists, and dieticians [2]. Dependent on the situation, goals of the treatment can be to obtain (i) a high locoregional control and survival rates in patients with limited disease, (ii) an increased survival in patients with advanced disease (improved locoregional control, reduced probability of distant metastasis, and second malignancies), (iii) an increased organ-function preservation in resectable and unresectable tumors, and (vi) an increased therapeutic ratio (cure/toxicity ratio) [3]. Single modality treatment is recommended for the patients with early-stage disease (stage I or stage II, approximately 40% of the patients with SCCHN) and combined modality treatment for patients with locally advanced disease. The combined modality treatment may include surgery followed by adjuvant radiotherapy or radiochemotherapy, concomitant radiochemotherapy (using conventional or alternative fractionation regimen), and induction chemotherapy followed by radiotherapy or radiochemotherapy [3, 4].

A precise understanding of prognostic factors is important to select the optimal treatment for the individual patient or to stratify patients for clinical trials or statistical analyses. In this retrospective single-institutional study, the role of potential prognostic factors was evaluated and compared in patients with squamous cell carcinoma of the oro- and hypopharynx after treatment with definitive radiotherapy/radiochemotherapy (dRT) versus surgery followed by postoperative radiotherapy (pRT).

## 2. Patients and Methods

Between 1992 and 2000, 288 patients with squamous cell carcinoma of the oropharynx or hypopharynx received a radiation therapy as definitive (*n* = 162) or as postoperative (*n* = 126) treatment.

Eligibility criteria for this retrospective single-institutional study were histologically proven squamous cell carcinoma of the oropharynx or hypopharynx, no distant metastasis or synchronous cancer at time of diagnosis, and definitive or postoperative radiotherapy with a minimum total dose of 60 Gy.

### 2.1. Radiation Therapy and Simultaneous Chemotherapy

One hundred and thirty-eight of 162 (85%) patients treated with definitive radiotherapy received a concomitant boost fractionation regimen, and 24 (15%) were treated with conventional fractionation (single fraction dose of 2 Gy, one fraction a day, five fractions a week). Two concomitant boost regimen were used: Regimen 1 consisted of a total dose of 66 Gy in five weeks with a daily fraction dose of 2 Gy and a concomitant boost of 1.6 Gy during the last two weeks (*n* = 76) and regimen 2 of a total dose of 69.9 Gy in 5.5 weeks with a daily fraction dose of 1.8 Gy and a concomitant boost of 1.5 Gy during the last 2.5 weeks (*n* = 62).

Ninety-four of 138 (68%) patients treated with concomitant boost fractionation regimen received a simultaneous chemotherapy as well as five (4%) patients treated with conventional fractionation.

The simultaneous chemotherapy consisted of 70 mg/m^2^ Carboplatin on days 1–5 and 29–33 (*n* = 73) or 70 mg/m^2^ Carboplatin and 600 mg/m^2^ 5-fluorouracil on days 1–5 and 29–33 (*n* = 26). Carboplatin was administered as a daily short-term intravenous infusion and 5-fluorouracil as an intravenous continuous infusion for 120 hours.

For the postoperative radiotherapy, only conventional fractionation without simultaneous chemotherapy was used (*n* = 123).

The radiotherapy was performed with opposed lateral fields for the upper neck and one anterior field for the lower neck using 6 MeV photons. Patients were treated in a thermoplastic mask for immobilization, and individual blocks were used to spare normal tissue where possible. After a dose of 30 to 36 Gy to the reference point, the spinal cord was spared out of the photon fields and the uninvolved posterior neck treated with electrons of selected energy according to CT findings with daily doses of 2.5 Gy five times a week to the prescribed total dose. Target volumes were defined on CT scans, and the dose was calculated to midplane. In selected patients, three-dimensional treatment planning was performed and conformal treatment techniques used.

### 2.2. Quantitative Determination of the Total Tumor Volume (TTV) from Digitised CT Scans

Pre-treatment CT scans of all patients were digitized with an automatic laser scanner (FIPS PLUS). The stored images were transferred to a personal computer. The macroscopic tumor shape (primary tumor and locoregional lymph node metastases) was defined in every CT slice (slice thickness 5 mm or 8 mm) using a drawing tool (software: photostyler). The number of pixels *n* enclosed by this contour was determined with a custom-shaped image processing program (software: Interactive Data Language). The area *A*_*i*_ of the *i*th slice was determined as *A*_*i*_ = pixel  size  (length)∗pixel  size  (width)∗*n*. The pixel size was determined using the scaling as given on the CT-hardcopy. The determined tumor area of each slice was multiplied with its slice thickness *d*_*i*_. The TTV was approximated by

(1) $$V_{\text{tumor}}\left(\text{c}{\text{m}}^{3}\right)=\overset{m}{\underset{i-1}{\sum }}A_{i}\left(\text{m}{\text{m}}^{2}\right)\times d_{i}\left(\text{mm}\right)/1000.$$

No interpolation between the CT slices was performed.

Repeated measurements using irregularly shaped tumor phantoms showed a difference between the reference volumes and CT-based volume measurements depending of the slice thickness (5 mm or 8 mm) of 1.4% to 4.5%.

### 2.3. Estimation of the Total Tumor Volume (TTV) Based on the Postoperative Histopathological Report

In addition to the quantitative tumor volumetry based on digitised pretreatment CT scans, TTV was estimated based on the postoperative histopathological report in patients treated with adjuvant radiotherapy. The TTV was approximated using the equation *V* = 4/3  *π*∗*a*∗*b*∗*c*, where *a*, *b*, and *c* represent the orthogonal maximal tumor diameters.

### 2.4. Statistical Analysis

The overall survival was defined as the time between the first day of the treatment and death of any cause. The overall survival was estimated using the Kaplan-Meier method, and treatment groups were compared using a two-sided log rank test. The locoregional failure-free survival was defined as the time between the first day of the treatment and a locoregional failure. The distant metastasis-free survival was defined as the time between the first day of the treatment and a distant failure. The locoregional failure-free survival and the distant metastasis-free survival were estimated by the cumulative incidence method, and treatment groups were compared using the Gray test [5, 6].

The simultaneous relationship of multiple prognostic factors to overall survival was assessed using Cox's proportional hazard regression analysis. The simultaneous relationship of multiple prognostic factors to locoregional failure-free or distant metastasis-free survival was assessed using the hazards of the cumulative incidence function model [7].

To estimate the reliability of the tumor volumetry, the TTV derived from digitised pretreatment CT scans was compared with the TTV derived from calculations based on tumor diameters provided by the postoperative histo-pathological report of the same patients. For the method comparison, the limits of agreement were estimated as described by Bland and Altman [8].

## 3. Results

The median follow-up time for the censored patients treated with definitive radiotherapy/radiochemotherapy was 28.5 months and for the patients treated with adjuvant postoperative radiotherapy 36.5 months. The 5-year overall survival for the patients treated with definitive radiotherapy/radiochemotherapy was 0.27 (95% CI 0.18–0.35) and for the patients treated with postoperative radiotherapy, 0.69 (95% CI 0.59–0.79).

The patient and treatment characteristics are demonstrated in Table 1. Patients treated with definitive radiotherapy/radiochemotherapy had a greater proportion of stage 4 tumors (96.9% versus 66.9%), a much larger median TTV (68.4 cm^3^ versus 21.2 cm^3^), and a lower proportion of preradiotherapy hemoglobin level ≤12 g/dL (17.6% versus 42.6%). The reduced Hb level was probably due to surgery-related blood loss. The performance status was not considered in this study because it was not consistently documented in the patient files.

### 3.1. Definitive Radiotherapy/Radiochemotherapy

**Figure 1:**
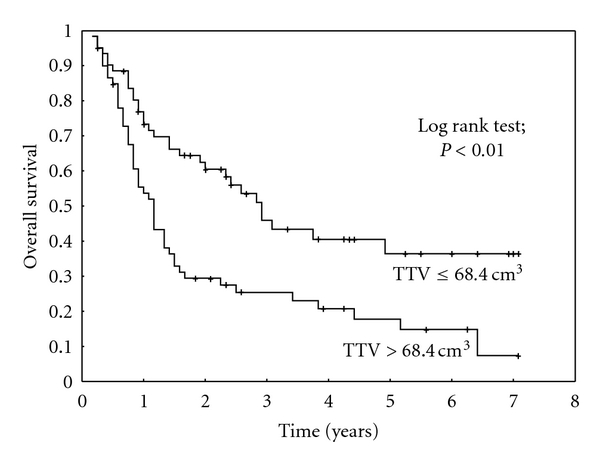
Impact of the total tumor volume (TTV) on overall survival in patients treated with definitive radiotherapy/radiochemotherapy.

**Figure 2:**
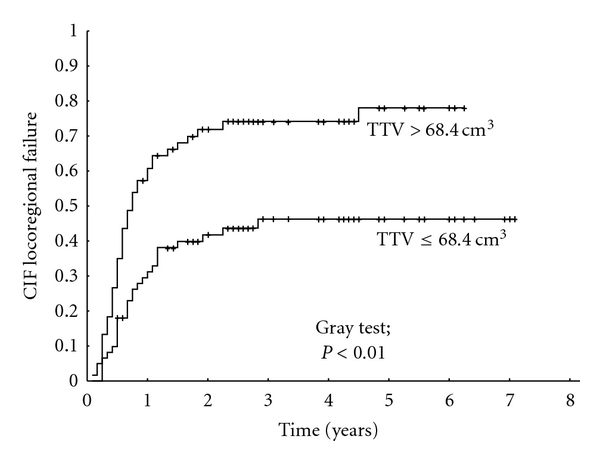
Impact of the total tumor volume (TTV) on locoregional control in patients with definitive radiotherapy/radiochemotherapy. CIF: cumulative incidence function.

On univariate analysis, the TTV (Figures 1 and 2) and the pre-radiotherapy hemoglobin level had a statistically significant impact on the overall survival and on the locoregional control of patients treated with definitive radiotherapy/radiochemotherapy. Simultaneous chemotherapy had a statistically significant effect on the overall survival and on the incidence of distant metastasis. In addition, there was a statistical trend of an association of simultaneous chemotherapy with the locoregional control (*P* = 0.08). The N-classification had a statistically significant impact on locoregional control but no significant impact on the overall survival or metastasis-free survival. The results of the univariate analysis are summarized in Table 2. On multivariate analysis, only the TTV and the simultaneous chemotherapy maintained their statistical significance (Table 3).

### 3.2. Postoperative Radiotherapy

**Figure 3:**
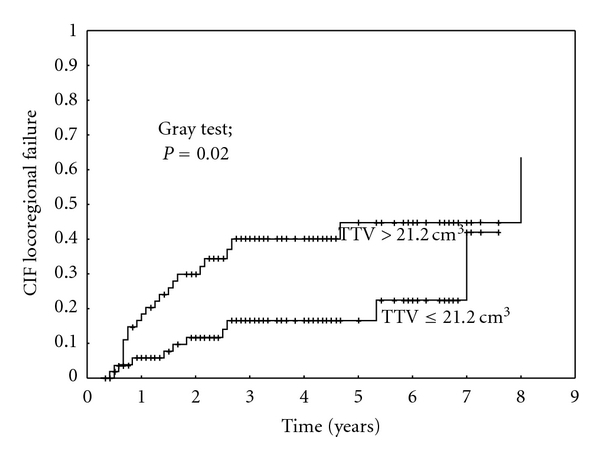
Impact of the total tumor volume (TTV) on locoregional control in patients treated with postoperative radiotherapy. CIF: cumulative incidence function.

On univariate analysis, the TTV had a statistically significant impact on the locoregional control (Figure 3). The N-classification had a statistically significant impact on the incidence of distant metastasis and a statistical trend on the locoregional control (*P* = 0.06). The results of the univariate analysis are summarized in Table 4. On multivariate analysis, only the TTV (*P* = 0.05) maintained its statistical significance (Table 5).

### 3.3. Tumor Volumetry Method Comparison

**Figure 4:**
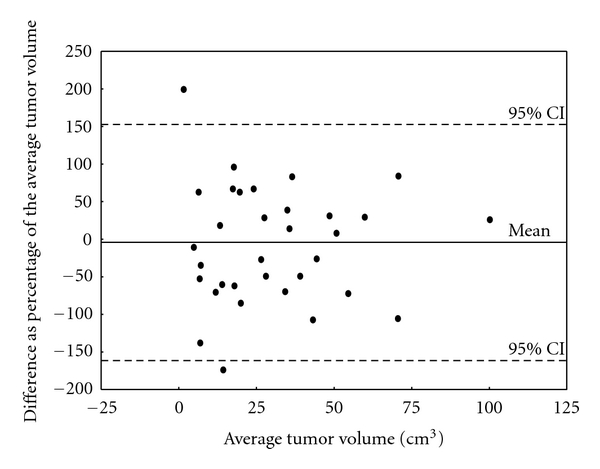
Difference as percentage of the average tumor volume of the quantitative CT-based tumor volumetry and estimation of the tumor volume based on the histological report plotted against their mean.

The TTV was estimated using quantitative tumor volumetry of digitized pre-treatment CT scans in all patients. In addition to the CT-based volumetry, in 34 patients, the TTV was also estimated based on tumor diameters reported in the histo-pathological report. The tumor volumes based on the two methods were compared to estimate the precision of the tumor volume measurements. The method comparison of the 34 patients showed that the 95% limit of agreement between the two total tumor volume measurements was approximately ±150% of the average total tumor volume measurement of both methods (Figure 4).

## 4. Discussion

This retrospective single institution analysis investigated possible prognostic factors of patients with squamous cell cancer of the oro- and hypopharynx treated with (i) definitive radiotherapy/radiochemotherapy or (ii) surgery followed by postoperative radiotherapy. The two patient groups were analysed separately because they differed considerably in respect to patient- and treatment-related characteristics and prognosis.

The tumor volume has been stated to be one of the most precise and most relevant predictor of radiotherapy outcome [9]. For patients with oro- and hypopharyngeal cancer treated with definitive radiotherapy or radiochemotherapy, the quantitative tumor volume was identified as significant prognostic factor in the majority of studies [10–20], and in few studies, as a prognostic factor of marginal [21, 22] or no significance [23].

In the multivariate analysis of our study, the total tumor volume had a statistically highly significant impact on the overall survival and locoregional control on patients treated with definitive radiotherapy/radiochemotherapy.

A new finding of our study is that the total tumor volume also had a statistically significant impact on the locoregional control in patients treated with surgery followed by postoperative radiotherapy. For this patient group, the total tumor volume was the only significant prognostic factor in the multivariate analysis. Our data suggest that the total tumor volume should be used to select patients for an intensified definitive or postoperative adjuvant treatment. Recent randomized studies have shown a significantly improved outcome of “high-risk” patients treated with adjuvant simultaneous radiochemotherapy compared to adjuvant radiotherapy alone [24, 25]. A comparative analysis of both studies revealed the extracapsular extension of tumor from neck nodes and/or microscopically involved surgical margins as significant clinical risk factors for poor outcome [26]. The quantitative total tumor volume was not considered in this analysis.

The only other significant prognostic factor in the multivariate analysis for patients treated with definitive radiotherapy in our study was the application of a simultaneous chemotherapy. The simultaneous chemotherapy was significantly associated with an improved overall survival and distant metastasis-free survival and showed a statistical trend of an improved locoregional control (*P* = 0.15). This observation is well in line with the literature. Randomized clinical trials [27–35] and meta-analyses [36, 37] have shown a significantly improved local control and survival with definitive simultaneous radiochemotherapy compared to definitive radiotherapy alone in patients with advanced squamous cell cancer of the head and neck.

Interestingly, in our study, the preradiotherapy hemoglobin level was a significant prognostic factor in the univariate analysis of patients treated with definitive radiotherapy/radiochemotherapy but lost its statistical significance in the multivariate analysis. If the total tumor volume was removed from the multivariate model, the pre-radiotherapy hemoglobin level retained its significance. Our data suggest that the total tumor volume is the stronger of both prognostic factors. A significant association of the pre-radiotherapy hemoglobin concentration with the treatment outcome after definitive radiotherapy/radiochemotherapy in the absence of quantitative tumor volume data has been reported by several studies in the literature [38–46].

For patients treated with postoperative radiotherapy, the pre-radiotherapy hemoglobin level showed no prognostic significance in our study. In the literature, differing findings are reported. One study found a significant impact of the pre-radiotherapy hemoglobin level on survival in patients with head and neck cancer treated with adjuvant radiotherapy [47]. Other studies evaluated the prognostic significance of the hemoglobin level at different time points during the treatment. In these studies, no prognostic significance was found for the pre-radiotherapy hemoglobin level, but for the hemoglobin level before surgery [48], after surgery [49, 50], duration of low hemoglobin level during the interval between surgery and radiotherapy [48], or for the difference of the hemoglobin concentration before and after adjuvant radiotherapy [51]. Several studies using pO_2_ histography have shown an impact of the tumor oxygenation on the survival of patients with head and neck cancer after radiotherapy [52–55], but no clear correlation was found between the tumor oxygenation by means of pO_2_ histography and the hemoglobin concentration [56, 57].

On multivariate analysis, the potential prognostic factors, gender, age, pre-radiotherapy hemoglobin level, tumor site, T- and N-, and R-status, RT-interruptions >5 days, and interval surgery-RT >32 days, showed no statistical significance in our study.

Other prognostic factors on multivariate analysis for locally advanced head and neck cancers reported by other studies were the performance status [45, 46], high-grade acute organ toxicity [58], or the UICC stage [59].

## 5. Conclusion

Our data suggest that the total tumor volume is the predominant prognostic factor in patients with squamous cell cancer of the oro- and hypopharynx treated with definitive radiotherapy/radiochemotherapy or surgery followed by postoperative radiotherapy. The total tumor volume should be used to identify high-risk patients and to stratify patients in clinical trials or statistical analyses.

## Figures and Tables

**Table 1:** Patient characteristics.

Characteristics	Definitive RT		Postoperative RT	
	*n*	%	*n*	%
Gender				
Female	17	10.5	19	15.1
Male	145	89.5	107	84.9
Age (years)				
≤56	87	53.7	72	57.1
>56	75	46.3	54	42.9
Hemoglobin level pre-RT (g/dL)				
≤12	27	17.6	46	42.6
>12	126	82.4	62	57.4
Tumor site				
Oropharynx	95	58.6	68	54.0
Hypopharynx	67	41.4	58	46.0
cT-Classification^#^				
1, 2	13	8.1	66	52.4
3, 4	148	91.9	60	47.6
cN-Classification^#^				
0, 1	14	3.7	50	39.7
2, 3	148	91.3	76	60.3
Stage				
≤3	4	3.1	42	33.3
4	157	96.9	84	66.7
Total tumor volume				
≤median*	52	43.0	52	46.8
>median*	69	57.0	59	53.2
R-Classification				
0	—	—	78	65.0
1	—	—	37	30.8
2	—	—	5	4.2
Fractionation regimen				
Conventional	24	14.8	126	100.0
Concomitant boost	138	85.2	0	0.0
Simultaneous radiochemotherapy				
Yes	99	61.1	0	0.0
No	63	38.9	126	100.0
RT interruptions >5 days				
Yes	36	22.2	24	20.0
No	126	77.8	96	80.0
Interval surgery-RT >32 days				
Yes	—	—	58	47.5
No	—	—	64	52.5

RT, radiation therapy; *median for definitive RT is 69.4 cm^3^, median for postoperative RT is 21.2 cm^3^.

**Table 2:** Definitive radiation therapy: univariate analysis of overall survival, locoregional control, and metastasis-free survival.

Factor	Overall survival		Locoregional failure		Metastasis	
	5y-survival	*P*	5y-CIF	*P*	5y-CIF	*P*
Gender						
Female	0.15 (95% CI 0.01–0.45)		0.60 (95% CI 0.50–0.68)		0.22 (95%0 CI 0.04–0.49)	
Male	0.28 (95% CI 0.19–0.37)	0.39	0.61 (95% CI 0.52–0.70)	0.52	0.33 (95% CI 0.24–0.42)	0.47
Age (years)						
≤56	0.27 (95% CI 0.17–0.39)		0.63 (95% CI 0.51–0.73)		0.34 (95% CI 0.23–0.45)	
>56	0.25 (95% CI 0.13–0.40)	0.43	0.63 (95% CI 0.46–0.76)	0.29	0.28 (95% CI 0.17–0.40)	0.61
Hemoglobin level pre-RT (g/dL)						
≤12	0.09 (95% CI 0.01–0.30)		0.82 (95% CI 0.56–0.93)		0.25 (95% CI 0.07–0.48)	
>12	0.30 (95% CI 0.20–0.40)	0.02	0.58 (95% CI 0.47–0.67)	0.01	0.33 (95% CI 0.23–0.42)	0.46
Tumor site						
Oropharynx	0.24 (95% CI 0.14–0.35)		0.69 (95% CI 0.56–0.78)		0.34 (95% CI 0.24–0.44)	
Hypopharynx	0.30 (95% CI 0.17–0.44)	0.70	0.54 (95% CI 0.39–0.67)	0.14	0.28 (95% CI 0.16–0.42)	0.32
T-Classification						
1, 2	0.44 (95% CI 0.13–0.72)		0.41 (95% CI 0.13–0.67)		0.25 (95% CI 0.05–0.51)	
3, 4	0.25 (95% CI 0.17–0.34)	0.34	0.65 (95% CI 0.55–0.73)	0.28	0.33 (95% CI 0.24–0.42)	0.70
N-Classification						
0, 1	0.52 (95% CI 0.19–0.77)		0.17 (95% CI 0.02–0.43)		0.16 (95% CI 0.02–0.41)	
2, 3	0.26 (95% CI 0.17–0.34)	0.17	0.66 (95% CI 0.56–0.74)	<0.01	0.33 (95% CI 0.24–0.41)	0.38
Total tumor volume (cm^3^)						
≤68.4	0.36 (95% CI 0.22–0.51)		0.46 (95% CI 0.33–0.59)		0.29 (95% CI 0.15–0.44)	
>68.4	0.18 (95% CI 0.09–0.30)	<0.01	0.78 (95% CI 0.62–0.88)	<0.01	0.37 (95% CI 0.24–0.50)	0.13
Simultaneous radiochemotherapy						
Yes	0.32 (95% CI 0.21–0.42)		0.57 (95% CI 0.46–0.67)		0.37 (95% CI 0.27–0.48)	
No	0.17 (95% CI 0.05–0.37)	0.03	0.73 (95% CI 0.53–0.85)	0.08	0.21 (95% CI 0.1–0.34)	0.04
RT interruptions >5 days						
Yes	0.15 (95% CI 0.01–0.43)		0.58 (95% CI 0.29–0.78)		0.28 (95% CI 0.13–0.44)	
No	0.28 (95% CI 0.19–0.38)	0.51	0.64 (95% CI 0.53–0.73)	0.83	0.33 (95% CI 0.23–0.42)	0.96

RT, radiation therapy; CIF, cumulative incidence function; *P*: *P* value.

**Table 3:** Definitive radiation therapy: multivariate analysis of overall survival, locoregional control, and metastasis-free survival.

Factor	Overall survival		Locoregional control		Metastasis-free survival	
	RR	*P*	RR	*P*	RR	*P*
Gender	1.34 (95% CI 0.62–2.91)	0.46	1.50 (95% CI 0.70–3.24)	0.30	0.79 (95% CI 0.15–4.07)	0.78
Age	1 (95% CI 0.63–1.59)	0.99	1.20 (95% CI 0.74–1.95)	0.45	0.96 (95% CI 0.47–1.98)	0.91
Hemoglobin level pre-RT	0.74 (95% CI 0.41–1.35)	0.33	0.74 (95% CI 0.42–1.31)	0.30	2.79 (95% CI 0.82–9.46)	0.10
Tumor site	1.24 (95% CI 0.76–2.04)	0.39	1.35 (95% CI 0.81–2.25)	0.24	1.40 (95% CI 0.62–3.15)	0.41
T-Classification	1.17 (95% CI 0.43–3.13)	0.76	0.75 (95% CI 0.22–2.58)	0.65	1.44 (95% CI 0.29–7.12)	0.66
N-Classification	1.14 (95% CI 0.38–3.43)	0.82	5.84 (95% CI 0.88–38.9)	0.07	2.28 (95% CI 0.21–25.1)	0.50
Total tumor volume	2.46 (95% CI 1.47–4.11)	<0.01	2.50 (95% CI 1.43–4.36)	<0.01	1.59 (95% CI 0.78–3.26)	0.20
Simultaneous radiochemotherapy	0.52 (95% CI 0.31–0.88)	0.01	0.68 (95% CI 0.40–1.16)	0.15	2.63 (95% CI 1.03–6.73)	0.04
RT interruptions >5 days	0.95 (95% CI 0.5–1.8)	0.88	0.77 (95% CI 0.36–1.66)	0.51	1.08 (95% CI 0.39–3.04)	0.88

RT, radiation therapy; RR, relative risk, *P*: *P*-value.

**Table 4:** Postoperative radiation therapy: univariate analysis of overall survival, locoregional control, and metastasis-free survival.

Factor	Overall survival		Locoregional failure		Metastasis	
	5y-survival	*P*	5y-CIF	*P*	5y-CIF	*P*
Gender						
Female	0.89 (95% CI 0.75–1.03)		0.53 (95% CI 0.10–0.85)		0.11 (95% CI 0.02–0.29)	
Male	0.66 (95% CI 0.54–0.77)	0.17	0.33 (95% CI 0.23–0.44)	0.56	0.21 (95% CI 0.14–0.30)	0.27
Age (years)						
≤56	0.72 (95% CI 0.60–0.84)		0.29 (95% CI 0.17–0.41)		0.21 (95% CI 0.12–0.31)	
>56	0.65 (95% CI 0.47–0.82)	0.87	0.45 (95% CI 0.27–0.61)	0.10	0.19 (95% CI 0.09–0.31)	0.85
Hemoglobin level pre-RT (g/dL)						
≤12	0.64 (95% CI 0.46–0.83)		0.41 (95% CI 0.20–0.61)		0.17 (95% CI 0.07–0.31)	
>12	0.69 (95% CI 0.55–0.84)	0.72	0.27 (95% CI 0.16–0.40)	0.42	0.23 (95% CI 0.13–0.35)	0.45
Tumor site						
Oropharynx	0.72 (95% CI 0.58–0.86)		0.44 (95% CI 0.27–0.59)		0.14 (95% CI 0.07–0.23)	
Hypopharynx	0.65 (95% CI 0.51–0.80)	0.35	0.28 (95% CI 0.16–0.41)	0.46	0.27 (95% CI 0.15–0.40)	0.30
T-Classification						
1, 2	0.69 (95% CI 0.54–0.83)		0.34 (95% CI 0.20–0.49)		0.19 (95% CI 0.11–0.30)	
3, 4	0.70 (95% CI 0.56–0.83)	0.80	0.37 (95% CI 0.23–0.52)	0.60	0.20 (95% CI 0.11–0.32)	0.94
N-Classification						
0, 1	0.72 (95% CI 0.54–0.89)		0.28 (95% CI 0.12–0.48)		0.10 (95% CI 0.04–0.21)	
2, 3	0.68 (95% CI 0.55–0.80)	0.24	0.40 (95% CI 0.27–0.53)	0.06	0.26 (95% CI 0.16–0.36)	0.03
R-Classification						
0	0.68 (95% CI 0.56–0.80)		0.37 (95% CI 0.23–0.50)		0.21 (95% CI 0.11–0.31)	
1, 2	0.69 (95% CI 0.59–0.79)	0.95	0.37 (95% CI 0.16–0.58)	0.81	0.20 (95% CI 0.07–0.33)	0.80
Total tumor volume (cm^3^)						
≤21.2	0.65 (95% CI 0.47–0.82)		0.23 (95% CI 0.09–0.37)		0.17 (95% CI 0.07–0.27)	
>21.2	0.61 (95% CI 0.45–0.76)	0.13	0.44 (95% CI 0.27–0.60)	0.04	0.26 (95% CI 0.14–0.39)	0.25
RT interruptions >5 days						
Yes	0.66 (95% CI 0.45–0.87)		0.51 (95% CI 0.26–0.72)		0.25 (95% CI 0.10–0.44)	
No	0.70 (95% CI 0.57–0.82)	0.22	0.27 (95% CI 0.17–0.39)	0.10	0.18 (95% CI 0.11–0.27)	0.26
Interval surgery-RT >32 days						
Yes	0.66 (95% CI 0.51–0.80)	0.50	0.45 (95% CI 0.28–0.60)	0.40	0.20 (95% CI 0.11–0.32)	0.79
No	0.70 (95% CI 0.54–0.85)		0.27 (95% CI 0.15–0.40)		0.20 (95% CI 0.11–0.32)	

RT: radiation therapy; CIF: cumulative incidence function; *P*: *P* value.

**Table 5:** Postoperative radiation therapy: multivariate analysis of overall survival, locoregional control, and metastasis-free survival.

Factor	Overall survival		Locoregional control		Metastasis-free survival	
	RR	*P*	RR	*P*	RR	*P*
Gender	0.34 (95% CI 0.04–2.74)	0.31	0.33 (95% CI 0.05–2.10)	0.24	0.63 (95% CI 0.05–8.47)	0.73
Age	1.11 (95% CI 0.49–2.54)	0.80	2.23 (95% CI 0.93–5.38)	0.07	1.05 (95% CI 0.36–3.04)	0.93
Hemoglobin level pre-RT	0.88 (95% CI 0.40–1.92)	0.75	0.79 (95% CI 0.32–1.93)	0.60	1.42 (95% CI 0.55–3.68)	0.47
Tumor site	0.98 (95% CI 0.42–2.26)	0.95	1.25 (95% CI 0.46–3.42)	0.66	0.65 (95% CI 0.25–1.72)	0.39
T-Classification	1.24 (95% CI 0.53–2.88)	0.62	1.85 (95% CI 0.77–4.42)	0.17	1.24 (95% CI 0.47–3.26)	0.66
N-Classification	1.17 (95% CI 0.45–3.09)	0.75	1.81 (95% CI 0.61–5.35)	0.28	2.25 (95% CI 0.72–7.01)	0.16
R-Classification	1.17 (95% CI 0.50–2.73)	0.72	0.79 (95% CI 0.34–1.87)	0.60	1.04 (95% CI 0.41–2.64)	0.94
Total tumor volume	1.77 (95% CI 0.76–4.14)	0.19	3.19 (95% CI 1.00–10.2)	0.05	1.03 (95% CI 0.37–2.85)	0.95
RT interruptions >5 days	1.42 (95% CI 0.57–3.50)	0.45	1.17 (95% CI 0.50–2.70)	0.72	1.15 (95% CI 0.31–4.31)	0.84
Interval surgery-RT >32 days	1.45 (95% CI 0.67–3.13)	0.35	1.92 (95% CI 0.81–4.54)	0.14	0.96 (95% CI 0.38–2.41)	0.93

RT: radiation therapy; RR: relative risk; *P*: *P* value.
